# Licorice root extract and magnesium isoglycyrrhizinate protect against triptolide-induced hepatotoxicity *via* up-regulation of the Nrf2 pathway

**DOI:** 10.1080/10717544.2018.1472676

**Published:** 2018-05-23

**Authors:** Qin-You Tan, Qian Hu, Sheng-Nan Zhu, Lu-Lu Jia, Juan Xiao, Hua-Zhen Su, Shao-Yuan Huang, Jing Zhang, Junfei Jin

**Affiliations:** a Clinical Pharmacy and Pharmacology Research Institute, The Affiliated Hospital of Guilin Medical University, Guilin, PR China;; b Laboratory of Hepatobiliary and Pancreatic Surgery, The Affiliated Hospital of Guilin Medical University, Guilin, PR China;; c China-USA Lipids in Health and Disease Research Center, Guilin Medical University, Guilin, PR China;; d Guangxi Key Laboratory of Molecular Medicine in Liver Injury and Repair, Guilin Medical University, Guilin, PR China

**Keywords:** Licorice root extract, MIG, Nrf2/ARE, L-02 cell, hepatotoxicity

## Abstract

Triptolide, the predominant biologically active component of the Chinese herb *Tripterygium wilfordii* Hook f., possesses numerous pharmacological activities, including anti-inflammatory, anti-fertility, anti-neoplastic, and immunosuppressive effects. However, toxicity and severe adverse effects, particularly hepatotoxicity, limit the clinical application of triptolide. Licorice root extract contains various bioactive compounds and is potent hepatoprotective. Magnesium isoglycyrrhizinate, a magnesium salt of the 18α-glycyrrhizic acid stereoisomer of glycyrrhizic acid, is used clinically in China to treat chronic viral hepatitis and acute drug-induced liver injury. The aim of this study was to investigate the role of the factor erythroid 2-related factor 2 pathway in the protective effects of LE and MIG against triptolide-induced hepatotoxicity. Hepatotoxicity models were established in L-02 cells and rats using triptolide, and the protective effects of LE and MIG were investigated *in vitro* and *in vivo*, respectively. LE and MIG significantly protected against triptolide-induced cytotoxicity. Additionally, triptolide decreased the mRNA and protein levels of Nrf2 and down-regulated Nrf2 target genes, including *UGT1A*, *BSEP*, and *MRP2*, while pretreatment with LE and MIG reversed these effects. Finally, Nrf2-involved antioxidant responses were activated in the presence of LE and MIG.

## Introduction

The diterpenoid compound triptolide (TP) is a major bioactive ingredient extracted from the widely used traditional Chinese medicinal herb *Tripterygium wilfordii* Hook f. (TWHF) (Xi et al., 2017). TP has been demonstrated to possess multiple biological activities, including anti-inflammatory, anti-neoplastic, anti-fertility, and immune modulation activities (Ziaei & Halaby, 2016). Tripterygium glycosides extracted from the root of TWHF containing TP has been used for clinical treatment of rheumatoid arthritis, immune complex nephritis, and systemic lupus erythematosus (Li et al., 2014). However, the clinical use of TP is limited due to its narrow therapeutic window and severe toxicity, including hepatotoxicity, nephrotoxicity, and reproductive toxicity (Wang et al., 2016b). Among these toxicities, hepatotoxicity is of most concern, as various TP-containing extracts of TWHF have been shown to lead to liver injury in animals and humans (Zhang et al., 2017).

One possible mechanism for TP-induced hepatotoxicity is damage from oxidative stress caused by reactive oxygen species (ROS) (Dan et al., 2015). The nuclear factor erythroid 2-related factor 2 (Nrf2), which has a basic leucine zipper structure, is an important redox-sensitive transcription factor that regulates the cellular oxidative stress response (Jeddi et al., 2017). Under normal physiological conditions, cytosolic Nrf2 is degraded through proteasome mediation by its binding partner Kelch-like ECH-associated protein 1 (Keap1) (Krajka-Kuźniak et al., 2015). Oxidative and electrophilic stresses cause dissociation of Nrf2 from Keap1, leading to Nrf2 translocation into the nucleus, where it dimerizes with small Maf-binding proteins and then with antioxidant response element (ARE) (Lacher et al., 2015; Sun et al., 2016). Nrf2 then activates many cytoprotective proteins and drug efflux transporters, such as uridine diphosphate glucuronosyltransferase (UGT), multidrug resistance-associated protein 2 (MRP2) and hemeoxygenase 1 (HO-1) (Yuan-Jing et al., 2016). As the most important mechanism underlying cellular protection against oxidative stress, the Nrf2/ARE signaling pathway, especially Nrf2, is regarded as a potential therapeutic target for preventing liver injury induced by oxidative stress.

Traditional medicinal licorice (*Glycyrrhiza glabra* L.) is obtained from the roots of *Glycyrrhiza uralensis* Fischer, *Glycyrrhiza glabra* L. or *Glycyrrhiza inflata* Batalin (Fabaceae), and the extract can enhance the effectiveness of other ingredients or reduce their toxicities (Gong et al., 2015; Chirumbolo, 2016). When treating rheumatoid arthritis, licorice can be combined with TWHF to mitigate the hepatotoxicity associated with TWHF (Cao et al., 2015); however, the hepatoprotective effect of licorice is not fully understood. Magnesium isoglycyrrhizinate (MIG), also known as tetrahydrate magnesium 18α, 20β-hydroxy-11-oxonorolean-12-en-3β-yl-2-O-β-D-glucopyranurosyl-α-D glucopyranosiduronate, is a magnesium salt of the 18-α glycyrrhizic acid stereoisomer in licorice root extract (LE) (Wang et al., 2017). MIG is an agent that protects hepatocytes. It is anti-inflammatory (He et al., 2010), protects liver cell membranes (Jiang et al., 2017), and improves liver function (Huang et al., 2014), and has been shown clinically to have hepatoprotective effects in cases of drug-induced liver injury, immune-mediated liver injury, and fatty liver (Tang et al., 2015). MIG has become a better candidate for treating inflammation and for hepatic protection than glycyrrhizin and β-glycyrrhizic acid due to its dramatic curative benefits and smaller number of adverse effects (Tang et al., 2015). It was recently reported that isoliquiritigenin and glycyrrhetinic acid are anti-oxidative and defend against TP-induced hepatotoxicity by activating Nrf2-associated HO-1 and NQO1 in HepG2 cells (Cao et al., 2016b); however, the hepatoprotective mechanisms of MIG remain to be elucidated.

In this study, the protective effects of LE and MIG against triptolide-induced hepatotoxicity, as well as the regulatory effects of LE and MIG on the Nrf2 pathway, were investigated.

## Materials and methods

### Chemicals and reagents

LE containing 11.8% sodium glycyrrhizinate was obtained from Xi’an Ruhong Biotechnology Co., Ltd. (Xi’an, China). MIG (purity >98%) was obtained from CHIATAI tianqing (Jiangsu, China). TP (purity >98%), rifampicin (RIF, purity >98%), and *tert*-butylhydroquinone (t-BHQ) were purchased from Aladdin Biochemical Technology Co., Ltd. (Shanghai, China). Dimethyl sulfoxide (DMSO), methyl thiazolyltetrazolium (MTT), penicillin-streptomycin solution, RIPA buffer, and 0.25% trypsin were purchased from Solarbio Life Science Co., Ltd. (Beijing, China). *UGT1A*, *BSEP*, Nrf2, and MRP2 antibodies were purchased from Abcam Biotechnology Co. (Milton, Cambridge, UK). An Electrochemiluminescence (ECL) plus kit was obtained from Bridgen Biology (Beijing, China). Other chemicals were of analytical grade and acquired from commercial suppliers.

### Cell culture

The human liver cell line L-02, obtained from Shanghai Cell Bank (Shanghai, China), was cultured in RPMI-1640 (Sigma-Aldrich, St. Louis, MO) supplemented with 10% (v/v) fetal bovine serum (Runsheng, Shanxi, China) and 1% penicillin-streptomycin solution. The cells were grown in a humidified incubator with 5% CO_2_ at 37 °C. LE, MIG, t-BHQ, and TP were dissolved in DMSO and stock solutions were stored at −20 °C. Reagents were freshly diluted to the indicated concentrations with culture medium before use. DMSO concentration in experimental conditions never exceeded 0.1% (v/v).

### Animals and treatments

Male Wistar rats (5 weeks old, weighing 180–220 g) were obtained from the Guilin Medical University Experimental Animal Centre (Guilin, China). The rats were housed in cages, under conditions of controlled temperature (24 ± 2 °C), humidity (50 ± 10%), a 12 h light/dark cycle, and had access to a standard diet and water *ad libitum*. The experiments were approved by the ethics committee for laboratory animal care in the Guilin Medical University according to Animal Ethics Procedures and Guidelines of the People’s Republic of China. Forty-two rats were randomly assigned to the following seven groups: (1) control; (2) TP (0.6 mg·kg^−1^); (3) MIG (13.5 mg·kg^−1^)+TP; (4) RIF (50 mg·kg^−1^)+TP; (5) low dose of LE (LLE) (120 mg·kg^−1^)+TP; (6) medium dose of LE (MLE) (240 mg·kg^−1^)+TP; and (7) high dose of LE (HLE) (480 mg·kg^−1^)+TP. Compounds were given *via* intragastric (i.g.) administration. Rat received MIG, RIF, or LE once daily for 7 consecutive days. An acute liver injury model was established by intragastric administration of TP (0.6 mg·kg^−1^) on the eighth day. After 18 h, rats were euthanized and required samples were collected.

### MTT assay

Cell viability was determined by MTT assay. First, the optimal concentration of TP or LE was determined. In brief, cells were seeded in a 96-well culture plate (5 × 10^3^ cells/well) overnight. Some cells were treated with TP (0.1, 1, 10, 20, 40, 80, 160, and 240 nmol·L^−1^) for 18 h and others were treated with LE (0, 30, 45, 60, 90, 180, 240, and 360 µg·mL^−1^) for 24 h. MTT (5 mg·mL^−1^, 20 μL/well) was added after 18 or 24 h and the cells were incubated for another 4 h after which the MTT was removed and DMSO (150 μL/well) was added. Finally, the absorbance was measured at 490 nm to identify the optimal concentration of TP or LE. Next, cells were seeded in a 96-well culture plate (5 × 10^3^ cells/well), incubated overnight and then pretreated with the optimal concentration of LE, MIG (30 µg·mL^−1^) or RIF (10 μmol·L^−1^) for 24 h and then treated with the optimal concentration of TP for 18 h. The MTT was then removed, DMSO added, the absorbance measured and the cell viability was calculated.

### Western blotting

Cells seeded in 6-well culture plates were pre-incubated with LE (30, 60, and 90 µg·mL^−1^), MIG (30 µg·mL^−1^), or RIF (10 μmol·L^−1^) for 24 h and then treated with the optimal concentration of TP for 18 h. The cells were subsequently lysed with RIPA buffer and the nuclear extracts were prepared using a Nuclear Extract Kit (Solarbio, China), according to the manufacturer’s recommendations. Equivalent amounts of protein were separated by 8% SDS-PAGE and transferred to a PVDF membrane. The membrane blocked in 5% nonfat milk in TBST for 1 h at room temperature and then incubated with primary antibodies at 4 °C overnight. The immunoblots were then incubated with a secondary antibody at room temperature. Finally, the antigen-antibody complex on the membrane was visualized using ECL plus and X-ray film. Total protein was extracted from rat liver tissue using RIPA lysis buffer and related proteins were determined *via* Western Blotting as above.

### Quantitative real-time PCR (qRT-PCR) analysis

Total RNA from cells or rat liver tissue was prepared using TRNzol Universal (TIANGEN, Beijing, China) according to the manufacturer’s protocol. Absorbance was measured at 260 and 280 nm to assess the quantity and purity of RNA. The cDNA was prepared from total RNA (1 μg) with a reverse transcriptase (RT) Primer Mix using the PrimeScript RT reagent Kit with gDNA Eraser (TIANGEN, Beijing, China) according to the manufacturer’s instructions. Supplementary Table 1 shows the primer sequences. Subsequent PCR amplification was carried out using a Bio-Rad CFX Manager 3.1 system (Bio-Rad, Hercules, CA) under the following conditions: 40 or 45 cycles at 95 °C for 5 s and at 60 °C for 20 s. Amplified products were monitored by measuring the increase of the dye intensity of the SYBR Green (TIANGEN, Beijing, China) that binds to double-strand DNA amplified by PCR. β-actin was used as an internal control.

### Immunofluorescence (IF) staining

L-02 cells were grown in a 24-well culture plate on glass coverslips for 24 h. Hepatocytes were pre-incubated with LE (30, 60, 90 µg·mL^−1^), MIG (30 µg·mL^−1^), or RIF (10 μmol·L^−1^) for 24 h and then treated with the optimal concentration of TP. Nrf2 expression in L-02 cells was detected 18 h later *via* immunohistochemical staining using the Nrf2 antibody. In brief, L-02 cells were fixed with 4% paraformaldehyde for 30 min, washed three times with PBS and then permeabilized with 0.5% Triton X-100 for 20 min. Samples were blocked in 1% BSA-supplemented PBS for 1 h and then incubated with anti-Nrf2 (1:200) primary antibody overnight at 4 °C. L-02 cells were then labeled with goat-anti-rabbit IgG (H + L)-FITC (1:200) for 1 h at room temperature. The slides were stained with phalloidin for 30 min and then mounted with DAPI for nuclei staining. The cells were washed twice with PBST after each step. Slides were stored at 4 °C, and then photographed under an invert fluorescent microscope equipped with 40x objectives.

### Hematoxylin and eosin (HE) staining, serum biochemical parameters, and antioxidant analysis

Serum samples were collected for assays of alanine transaminase (ALT), aspartate transaminase (AST), malondialdehyde (MDA), superoxide dismutase (SOD), and glutathione (GSH). Livers from rats were collected for tissue processing and staining. The specimens were immersed in a formaldehyde solution (37–40% formaldehyde/PBS: 1:9) for 24 h. After fixation, the specimens were transferred to 70% ethanol until use. The specimens were processed through different grades of alcohol, cleared in Van-clear (substitute for xylene) and embedded in paraffin. Paraffin-embedded specimens were then cut into 3 mm sections and stained with hematoxylin and eosin for overall morphological evaluation.

### Statistical analysis

Results are presented as mean ± standard deviation (SD) and were analyzed by one-way ANOVA, followed by Tukey’s test using SPSS version 19.0 (SPSS, Chicago, IL). A *p* value less than .05 was considered statistically significant.

## Results

### Effects of LE, MIG, and TP on liver cell viability

**Figure 1.**
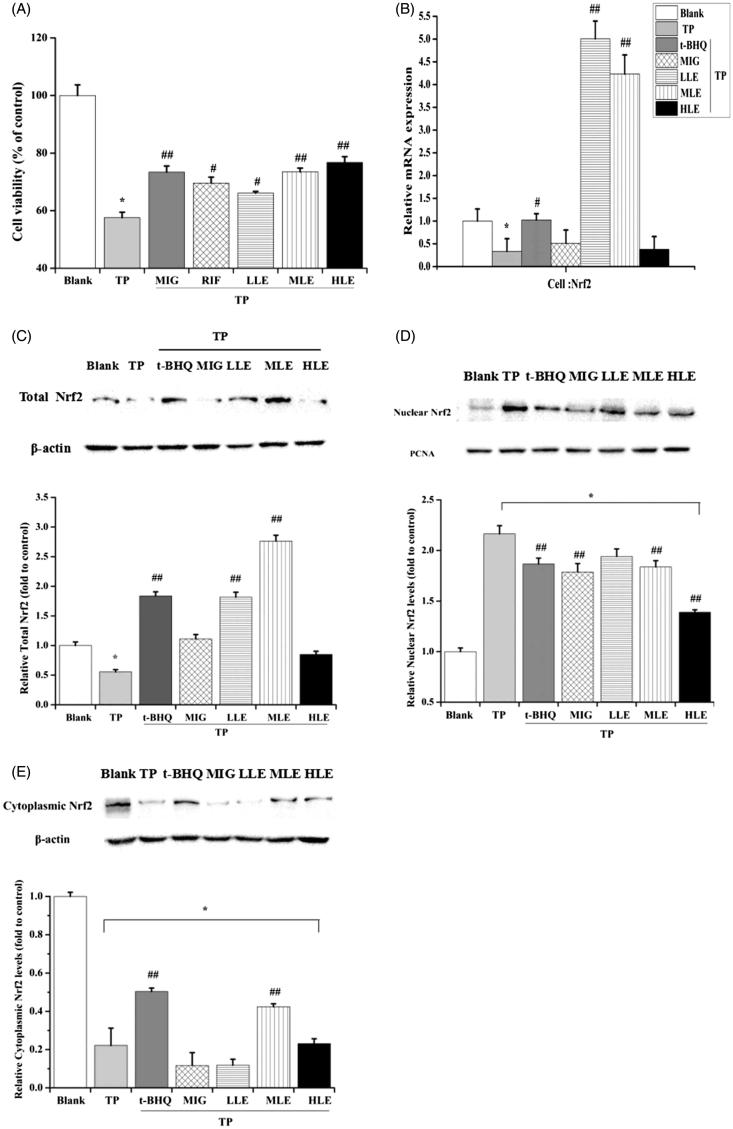
The effect of LE and MIG on Nrf2 in L-02 cells exposed to TP. (A) Cells were pretreated with MIG, RIF or various concentrations of LE for 24 h, then exposed to TP for 18 h, finally cell viability of L-02 cells was measured by MTT assay, (*x* ± *s*, *n* = 6). Cells were treated with t-BHQ, MIG, or different concentrations of LE for 24 h, and then exposed to 80 nM TP for 18 h, finally, Nrf2 mRNA expression (B), Nrf2 total protein (C), nuclear Nrf2 (D) and cytoplasmic Nrf2 (E) in L-02 cells were determined. (*x* ± *s*, *n* = 6). *p < .05 versus control; #*p* < .05 versus TP group; ##*p <* .01 versus TP group.

To determine the optimal LE concentration, L-02 cells were treated with tested drugs at indicated concentration and cell viability was measured using an MTT assay. As shown in Figure S1(A), LE increased cell viability in dose-dependent manner. Thus, the low dose (30 µg·mL^−1^), middle dose (60 µg·mL^−1^), and high dose (90 µg·mL^−1^) of LE were chosen for the following experiments. After exposure to TP for 18 h, the relative cell survival rate decreased dose-dependently (Figure S1(B)) (*p* < .05) suggesting that TP is toxic to liver cells. Based on this, duration of 18 h and a TP concentration of 80 nM were selected for the following experiment. As shown in Figure 1(A), the viability of cells pre-incubated with MIG was significantly higher than the viability of cells in the TP group, and LE increased the cell viability in dose-dependent manner (*p* < .05) indicating that both LE and MIG protected liver cells from toxicity induced by TP.

#### Effect of LE, MIG, and TP on Nrf2 in L-02 cells

Since TP-induced hepatotoxicity is related to ROS and the Nrf2/ARE signaling pathway is very important for regulating oxidative stress, we hypothesized that Nrf2 is involved in the hepatotoxicity caused by TP. First, Nrf2 mRNA expression was measured to determine the effect of TP on Nrf2. TP significantly decreased Nrf2 mRNA expression, which was inhibited by both LE and t-BHQ (Figure 1(B)). Consistent with the mRNA result, there was a pronounced suppression of Nrf2 total protein with TP treatment (Figure 1(C)). The total level of Nrf2 recovered with LE, but not MIG, treatment in the presence of TP (Figure 1(C)). Since Nrf2 initiates the anti-oxidative process in the nucleus, the impact of LE, MIG, and TP on Nrf2 translocation was also examined. Nuclear and cytoplasmic Nrf2 were detected by Western blot. TP promoted the Nrf2 translocation into the nucleus, however, LE and MIG did the opposite (Figure 1(D,E)); t-BHQ was utilized as a positive control. These results indicate that the promotion of Nrf2 translocation into the nucleus by TP is involved in TP-induced hepatotoxicity and that LE and MIG may reduce this hepatotoxicity by reducing Nrf2 translocation into nucleus.

### Immunofluorescent multi-staining analysis of Nrf2 in L-02 cells

**Figure 2.**
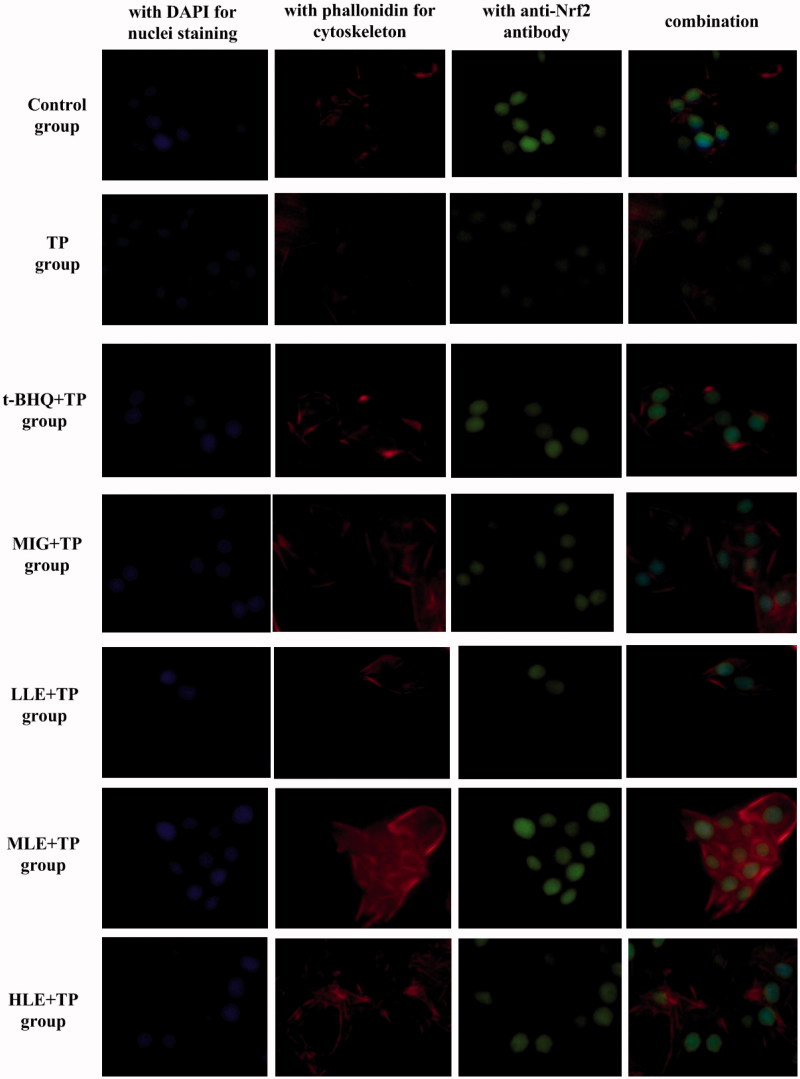
Nuclear translocation of Nrf2 in hepatocytes as demonstrated by immunofluorescent multi-staining. Immunoreactivity and colocalization of Nrf2 protein were analyzed in cells pretreated with t-BHQ, MIG, or different concentrations of LE for 24 h and then exposed to 80 nM TP for 18 h. Blue indicates nuclei (DAPI staining), red indicates cytoskeleton (phalloidin), and green indicates Nrf2. Original magnification 400× Bar =20 μm.

To further determine Nrf2 expression and nuclear translocation, L-02 cells were treated as indicated and then subjected to immunofluorescent assay using specific antibodies and then analyzed with an inverted fluorescent microscope. DAPI nuclear stain (blue) and anti-Nrf2 antibody stain (green) almost overlap in the cells of other groups, compared to control group cells (Figure 2). This indicates that treatment with TP induced nuclear translocation of Nrf2 in the L-02 cells. L-02 cells treated with LE (30, 60, and 90 µg·mL^−1^) or MIG at (30 µg·mL^−1^) for 24 h had more total Nrf2 (green) in the presence of TP compared to cells treated with TP alone (Figure 2).

#### Effects of LE, MIG, and TP on MRP2, UGT1A, and BSEP in L-02 cells

**Figure 3.**
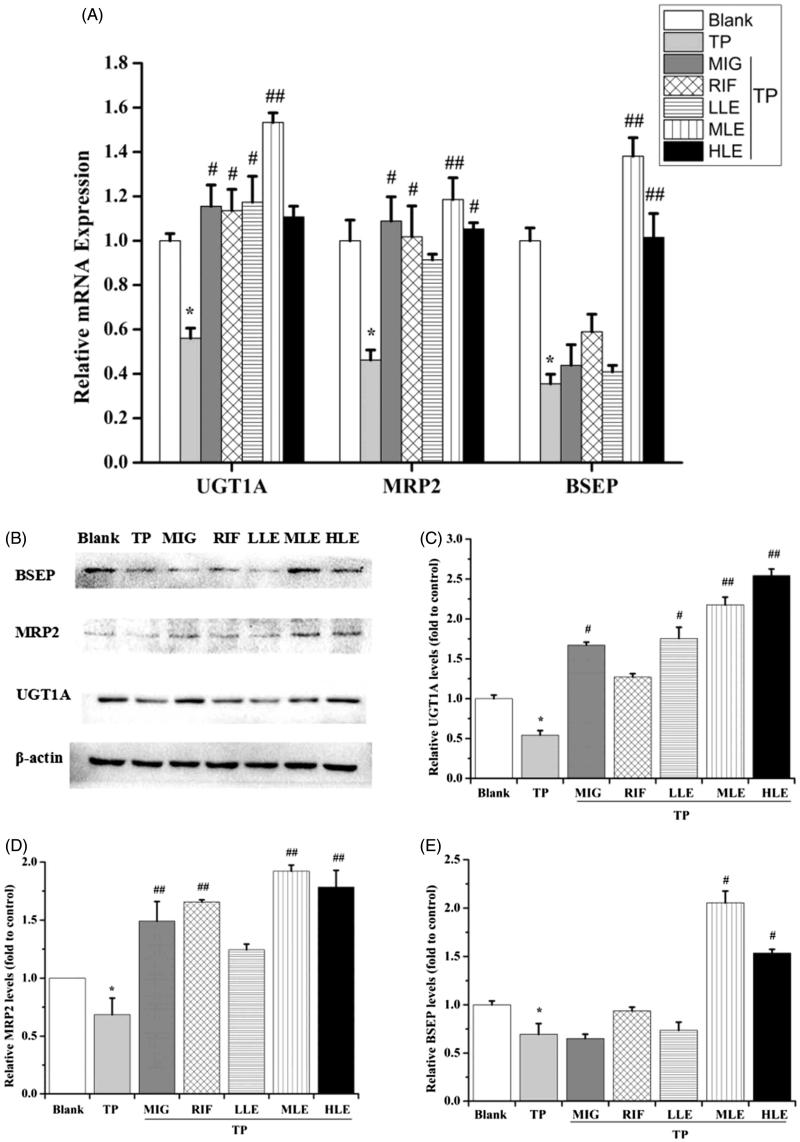
The effects of LE and MIG on UGT1A, MRP2, and BSEP in L-02 cells exposed to TP. Cells were treated with MIG, RIF or various concentrations of LE for 24 h, and then exposed to 80 nM TP for 18 h. The mRNA expression (A) and protein level (B) of *UGT1A*, *MRP2*, and *BSEP* were measured. Western blot gray value of *UGT1A* (C), *MRP2* (D), and *BSEP* (E) from (B) are shown (*x* ± *s*, *n* = 6). *p < .01 versus control; #*p* < .05 versus TP group; ##*p* < .01 versus TP group.

To investigate the role of Nrf2 in the protection provided by LE against liver toxicity caused by TP, the expression of Nrf2 downstream proteins, including UGT1A, MRP2, and BSEP, were measured in L-02 cells. Quantitative real-time PCR (qRT-PCR) and Western blot showed that TP significantly decreased the mRNA and protein levels of UGT1A, MRP2, and BSEP (Figure 3), LE and MIG reversed the effect of TP in a dose-dependent manner. Taken together, these data indicate that the hepatoprotective effect of LE against TP-induced liver toxicity may be partial *via* the Nrf2 signaling pathway, which enhances detoxification and antioxidant capacities of cells.

### Effects of LE, MIG, and TP on serum biochemical indicators in rats

As shown in Table 1, the serum activities of AST and ALT in the TP-treated group increased significantly (*p* < .01) compared with the blank group, suggesting that the rat model of TP-induced hepatotoxicity was successful. Administration of LLE significantly reduced the elevation in serum AST and ALT activity induced by TP (*p* < .01). MIG also diminished the increase in serum ALT activity (*p* < .05). TP significantly inhibited the serum activities of SOD (*p* < .05) and GSH-Px (*p* < .01) and increased the serum level of MDA (*p* < .01). Conversely, treatment with LLE resulted in a significant increase in the amount of GSH-Px (*p* < .05) and a significant decrease in the level of MDA (*p* < .01).

**Table1.** Comparison of serum ALT, AST, MDA, SOD, and GSH-Px levels among various groups (*x* ± *s*, *n* = 6).

Group	ALT (U/L)	AST (U/L)	MDA (nmol/L)	SOD (U/ml)	GSH-Px (μmol/L)
Blank	33.81 ± 6.48**	68.75 ± 5.92**	5.31 ± 0.16**	276.90 ± 22.77*	1777.50 ± 169.07**
TP	52.44 ± 9.91	87.58 ± 9.93	6.31 ± 0.44	248.74 ± 15.96	1455.48 ± 150.67
MIG + TP	39.45 ± 7.49*	77.42 ± 9.22	6.14 ± 0.85	233.90 ± 18.29	1470.00 ± 143.43
RIF + TP	33.97 ± 8.11**	73.23 ± 8.51*	5.98 ± 0.30	233.22 ± 15.76	1529.76 ± 157.09
LLE + TP	31.12 ± 3.66**	66.20 ± 4.53**	5.56 ± 0.28**	261.38 ± 9.70	1622.90 ± 52.30*
MLE + TP	40.63 ± 3.72*	77.98 ± 6.85	6.11 ± 0.61	248.40 ± 12.42	1431.05 ± 95.69
HLE + TP	35.31 ± 5.14**	65.13 ± 6.30**	5.75 ± 0.18*	256.32 ± 6.67	1620.24 ± 117.91

* *p* < .05.

** *p* < .01 versus TP group.

#### Effects of LE, MIG, and TP on histopathological variations in the liver

**Figure 4.**
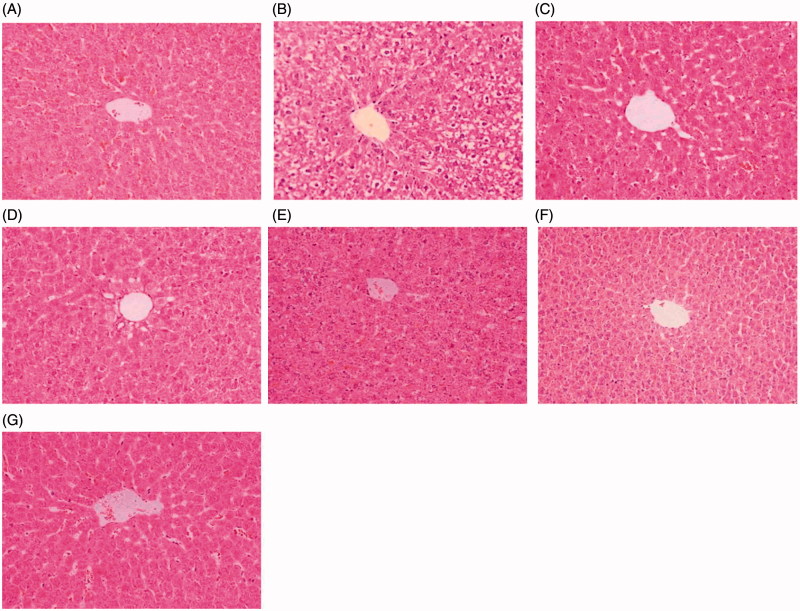
Photomicrographs of HE-stained liver sections. Tissues were divided into 3-mm sections and stained with hematoxylin and eosin for light microscopic analysis. The photomicrographs are of livers obtained from (A) Control group, (B) TP group, (C) MIG + TP group, (D) RIF + TP group (E) LLE + TP group, (F) MLE + TP group, (G) HLE + TP group.

Histopathological analysis of the livers of rats under indicated treatments is shown in Figure 4. In the control group, the livers exhibited normal hepatic cells with clear nuclei and well-preserved cytoplasms (Figure 4(A)). Livers of rats in the TP group showed significant histopathological changes, including apparent severe hepatocellular hydropic degeneration and necrosis (Figure 4(B)). The levels of hepatocellular hydropic degeneration and necrosis were slightly decreased in the MIG + TP, RIF + TP, and LLE + TP groups (Figure 4(C–E)) and were significantly decreased in the MLE + TP and HLE + TP groups (Figure 4(F–G)). Taken together, these results indicate that LE prevented TP-induced liver injury in rats.

#### Effects of LE, MIG, and TP on Nrf2 in rats

**Figure 5.**
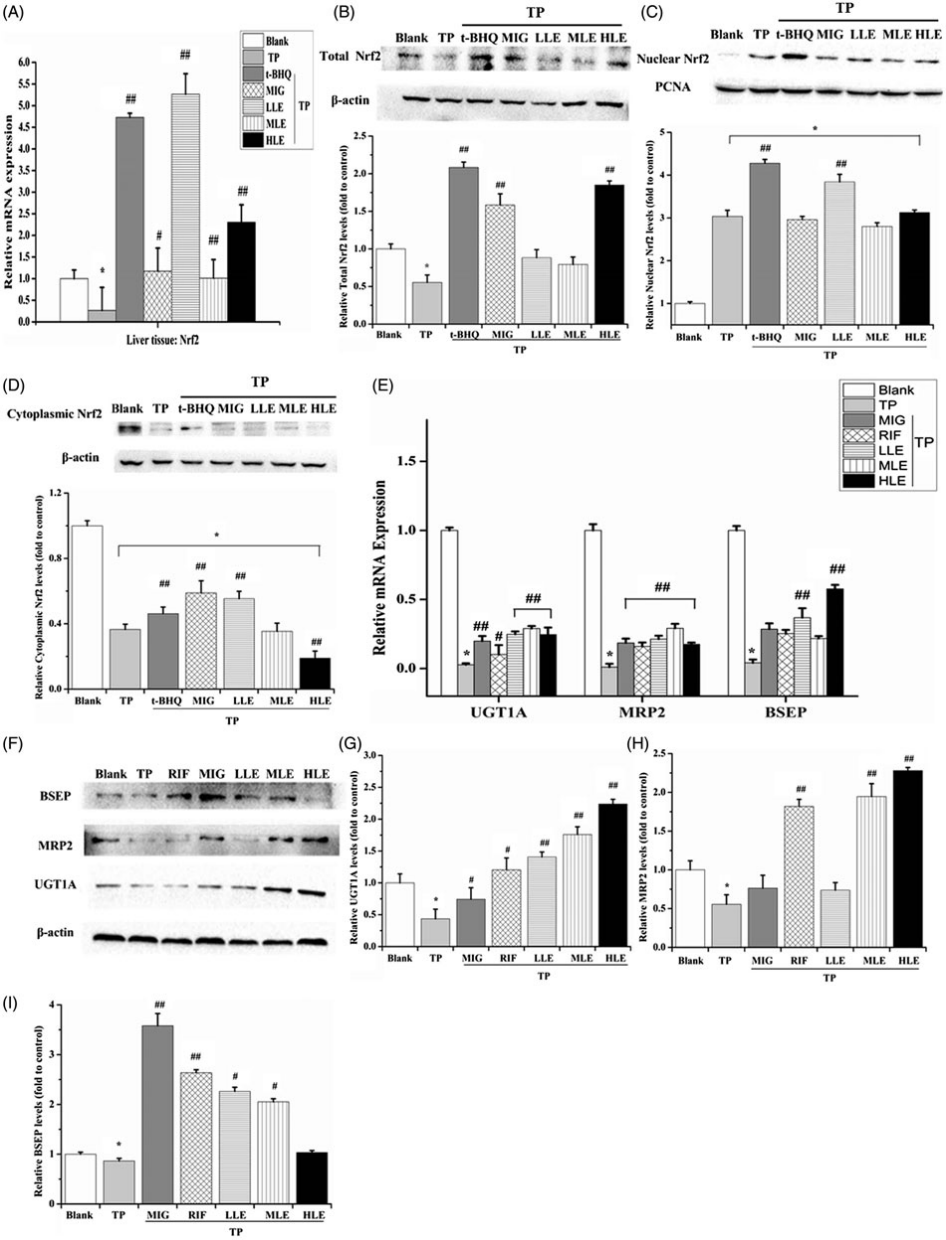
The effects of LE and MIG on Nrf2 pathway in rat liver tissues exposed to TP. Rats were treated with t-BHQ, MIG, and different concentrations of LE (for A–D); or MIG, RIF, and different concentrations of LE (for E–I) for 24 h and were then exposed to 80 nM TP for 18 h, finally liver tissues were collected. Nrf2 mRNA expression (A), Nrf2 total protein level (B), nuclear Nrf2 (C), cytoplasmic Nrf2 (D), mRNA expression (E) and protein (F) of UGT1A/MRP2/BSEP were detected in rat liver tissues. (*x* ± *s*, *n* = 6) *p < .01 versus control; #*p* < .01 versus TP group; ##*p* < .05 versus TP group. The western blot gray value of UGT1A (G), MRP2 (H), and BSEP (I) from (F) were measured (x ± s, *n* = 6). All results are expressed as the *p < .01 versus control; #*p* < .05 versus TP group; ##*p* < .01 versus TP group.

To validate the variation in protein level and translocation of Nrf2, Nrf2 mRNA expression, total Nrf2 protein level, nuclear Nrf2, and cytoplasmic Nrf2 in the rat liver were determined by RT-PCR and Western blot. Similar to L-02 cells, TP decreased mRNA level and Nrf2 total protein and treatment with LE and MIG reversed these effects (Figure 5(A,B)). However, LE and MIG did not suppress the Nrf2 nuclear translocation induced by TP (Figure 5(C,D)).

#### Effects of LE, MIG, and TP on MRP2, UGT1A, and BSEP in rats

To verify the role of the Nrf2 signaling pathway in the livers of rats under the indicated treatments, the mRNA (Figure 5(E)) and protein (Figure 5(F–I)) level of MRP2, UGT1A, and BSEP were measured in the liver tissues. TP decreased MRP2, UGT1A, and BSEP; however, LE and MIG inhibited this TP-induced decrease, indicating that LE and MIG activated Nrf2-involved antioxidant response by up-regulating downstream genes in the livers of TP-treated rats.

## Discussion

TP, one of the main active components in the Chinese herb TWHF, has been widely used to treat various diseases, including rheumatoid arthritis, nephritic syndrome, and lupus (Hao et al., 2018). However, multiple organ toxicity due to TP, especially hepatotoxicity, greatly limits its clinical application (Hai-juan et al., 2016; Wei et al., 2017). Thus, TWHF is frequently used in combination with other Chinese herbs, such as *G. uralensis* Fisch (licorice) root extract. LE has a variety of beneficial biological properties. It is anti-inflammatory, anti-bacterial, and has anti-tumor activity (Cao et al., 2016a; Fukuchi et al., 2016). MIG is the magnesium salt of the 18α-glycyrrhizic acid stereoisomer of glycyrrhizic acid and is used to treat chronic viral hepatitis and acute drug-induced liver injury. In this study, TP was found to cause damage to liver cells; LE and MIG exerted cytoprotective effect in which Nrf2 signaling pathway is involved.

Nrf2 is a cellular sensor of chemically induced oxidative stress and functions to restore homeostasis by up-regulating antioxidants, xenobiotic metabolism, and other cytoprotective enzymes (Gallorini et al., 2015). Recently, it was found that Nrf2 plays a protective role due to its involvement in the coordinated induction of phase II enzymes and phase III drug transporters (Park et al., 2016). UGT is specifically expressed in hepatic and extrahepatic tissues, which are critical in endobiotic and xenobiotic metabolisms (Filopanti et al., 2014). UGT1A transforms lipophilic molecules into more polar forms, thus facilitating its subsequent elimination *via* bile, feces, and urine (Wang et al., 2016a). MRP2 participates in excretion of chemicals into bile, especially GSH-, glucuronide-, and sulfate-conjugated metabolites (Qu et al., 2017). Bile salt export pump (BSEP/ABCB11) is also expressed in the canalicular membrane. BSEP is the major transporter of bile salts secreted by liver cells into the bile, the variation, and inhibition of which are connected with cholestasis and drug-induced liver injury (Chen et al., 2016). In addition, mutations in the BSEP gene are associated with progressive familial intrahepatic cholestasis type II; therefore, BSEP plays an essential role in the biliary excretion of bile acids.

Nrf2 has become an intensely researched topic in the study of liver diseases and is expected to become a potential target for their treatment, such as liver injury and hepatic fibrosis. With the aid of phase II metabolic enzymes and III transporters, the toxic efflux of intracellular to extracellular substances will promote the elimination of toxic substances. Detoxification plays a role in organ protection, and relevant research is currently being conducted.

Recently, it was reported that LE induced NQO1, Nrf2, and UGT and ARE-luciferase activity in Nrf2 wild type (Nrf2^+/+^) mice. However, this effect was not observed in Nrf2-knockout (Nrf2^−/−^) mice (Wu et al., 2011). This study provides evidence that LE strongly activates the Nrf2/ARE/anti-oxidative stress signaling pathways. Therefore, we hypothesize that LE strongly regulates the Nrf2/ARE/anti-oxidative stress signaling pathways, phase II detoxifying enzymes and drug transporter induction, which would contribute to pharmacological effects that promote overall health and inhibit diseases, including cancer. A recent study found that tanshinone II A could activate the Nrf2/ARE pathway and protect mice from acute liver injury caused by TP (Guan et al., 2013). In addition, Nrf2 may also play a protective role against NRK-52E cytotoxicity induced by *T. wilfordii* (Li et al., 2012). Based on the literature and previous studies, it is speculated that *G.uralensis* Fisch. plays a protective role in hepatic toxicity induced by *T. wilfordii* through activation of the Nrf2 signaling pathway. At present, additional research is being conducted.

MRP2 is an important transporter that mediates the excretion of cytotoxic substances and carcinogenic terminals and plays an important role in detoxification and prevention in the body. Studies have shown that the physiological activity and expression level of MRP2 will affect drug efficacy and toxicity of methotrexate in patients with hyperbilirubinemia (Liu et al., 2015). In addition, researchers are attempting to alleviate irinotecan toxicity, a chemotherapeutic drug, by modulating MRP2 expression and activity (Yokooji, 2013). In Bsep-KO mice, marked up-regulation of potential compensatory bile acid handling transporters, such as MRP2/MRP3, as well as down-regulation of the bile acid uptake transporter sodium taurocholate co-transporting polypeptide (Ntcp) suggest a pattern of expression consistent with the prevention of intrahepatic accumulation of bile acids (Lam et al., 2005). LE can significantly induce the expression of MRP2, which is conducive to rapid excretion of toxic substances in the body, to prevent and control the accumulation of poisons, which reduces toxic exposure and increases the ability of the body to detoxify itself. LE activates a series of detoxification-related genes, up-regulates the body’s defense system and improves the body’s ability to combat toxic substances.

In this study, results from experiments with cells and animals confirmed that LE and MIG can activate the Nrf2/ARE cell-signaling pathway. LE up-regulates Nrf2 expression and its downstream phase II detoxification enzyme, UGT1A, and the mRNA expression of the transporters BSEP and MRP2, suggesting that LE plays a role in detoxification by accelerating the excretion of toxins from the body by activating the Nrf2/ARE signaling pathway and inducing the expression of protective genes, such as the downstream phase II detoxification enzymes and drug transporters. Nrf2, MRP2, UGT1A, and BSEP levels were down-regulated after TP treatment, while LE and MIG pretreatment rescued the expression of Nrf2 in both L-02 cells and liver tissue. It has been reported that 18β-glycyrrhetinic, a main active ingredient obtained from licorice, attenuates oxidative stress and inflammation *via* up-regulating Nrf2/ARE signaling (Lam et al., 2005). Together with the finding that the α, β-unsaturated carbonyl group modifies specific cysteine residues of Keap1 (Abd El-Twab et al., 2016), LE and MIG were confirmed to activate Keap1/Nrf2/ARE pathway. Although numerous studies have demonstrated that LE and MIG activate the Nrf2 pathway, this study combined LE or MIG with TP to assess their effects on reducing TP-induced hepatotoxicity.

Nuclear Nrf2 activates the downstream genes responsible for oxidative stress regulation. However, TP promotes Nrf2 translocation into the nucleus, which then down-regulates Nrf2 target genes. This phenomenon might be due to an uncertain relationship between nuclear Nrf2 and its downstream pathway. Furthermore, the effects of LE and MIG on the location of Nrf2 in the liver were not consistent with results in L-02 cells, indicating the possibility that dynamic changes play a part in the protective effect of these two treatments. Moreover, since LE and MIG have other biological targets, the involvement of other pathways in the protective effect of LE and MIG in the presence of TP cannot be excluded. Further studies investigating the protective activity of LE and MIG against TP-induced-toxicity in an Nrf2^−/−^animal are needed.

The phase II detoxification enzyme UGT1A plays an important role in the removal of toxic metabolites and in excretion, together with the transporters MRP2 and BSEP. These may also be regulated by the Nrf2/ARE signal transduction pathway, thus strengthening the toxin efflux. Under stress conditions, such as poisoning, the oxidative stress enzymes NQO1, HO-1, SOD, and CAT regulate redox balance, change the system from oxidative stress to equilibrium, and return the body to a normal physiological state. Liver function was assessed by measuring the activities of serum biomarkers (i.e. ALT, AST, ALP, and LDH). The increased levels of ALT, AST, and ALP are conventional indicators of hepatocellular necrosis (Sechanie & Moshtaghie, 2016). To some extent, LDH can be treated as an indicator of liver injury, although LDH isoenzymes are not liver-specific (Zhu et al., 2016). In this study, hepatotoxicity of TP and the protective roles of LE and MIG were validated by variations in serum levels of ALT, AST, MDA, SOD, and GSH-Px. In addition, histopathological analyses of rat liver tissues were consistent with the biochemistry assays and L-02 cell morphological analyses.

In summary, LE and MIG can activate the Nrf2 pathway, increasing the mRNA and protein expression of Nrf2 target genes and reducing TP-induced hepatotoxicity in L-02 cells and rats. This study provides new insights into the development of strategies to prevent or alleviate TP-induced hepatotoxicity.

## Conclusion

LE and MIG effectively attenuated TP-induced oxidative stress, and the Nrf2 pathway was involved in this regulation. Further investigations *in vitro* and *in vivo* are needed. These results provide new strategies to repress oxidative stress-induced by TP. Moreover, this study implies that the Nrf2 pathway may present a new biological target, and that LE and MIG might be candidates for the prevention of drug-induced hepatotoxicity, partly *via* the Nrf2 pathway.
